# Mechanics of serotonin-producing human entero-endocrine cells

**DOI:** 10.1016/j.mbm.2024.100044

**Published:** 2024-02-08

**Authors:** Tom M.J. Evers, Joep Beumer, Hans Clevers, Alireza Mashaghi

**Affiliations:** a Medical Systems Biophysics and Bioengineering, Leiden Academic Centre for Drug Research, Faculty of Science, Leiden University, the Netherlands; b Laboratory for Interdisciplinary Medical Innovations, Centre for Interdisciplinary Genome Research, Faculty of Science, Leiden University, the Netherlands; c Hubrecht Institute, Royal Netherlands Academy of Arts and Sciences (KNAW) and UMC Utrecht, Utrecht, the Netherlands; d Oncode Institute, Hubrecht Institute, Utrecht, the Netherlands; e The Princess Maxima Center for Pediatric Oncology, Utrecht, the Netherlands

**Keywords:** Cell mechanics, Gut, Organoid, Serotonin, Optical tweezers

## Abstract

The gastrointestinal (GI) tract's primary role is food digestion, relying on coordinated fluid secretion and bowel movements triggered by mechanosensation. Enteroendocrine cells (EECs) are specialized mechanosensitive cells that convert mechanical forces into electrochemical signals, culminating in serotonin release to regulate GI motility. Despite their pivotal role, knowledge of EEC mechanical properties has been lacking due to their rarity and limited accessibility. In this brief report, we present the first single-cell mechanical characterization of human ECCs isolated from healthy intestinal organoids. Using single-cell optical tweezers, we measured EEC stiffness profiles at the physiological temperature and investigated changes following tryptophan metabolism inhibition. Our findings not only shed light on EEC mechanics but also highlight the potential of adult stem cell-derived organoids for studying these elusive cells.

## 1 Introduction

The primary function of the gastrointestinal (GI) tract is to digest food, a process that depends on coordinated fluid secretion and bowel movement in response to luminal mechanical stimuli. The GI tract detects such stimuli through the process of mechanosensation.^1^ Mechanosensation depends on specialized mechanosensitive cells that convert mechanical force into electrochemical signals using mechanoreceptors.^2^ An important example includes the epithelial enteroendocrine (EEC) cells, and in particular enterochromaffin cells, which release the neurotransmitter serotonin in response to mechanical stimulation.^3^, ^4^, ^5^ The secreted serotonin, in turn, is transduced (mechanotransduction) and then relayed by specialized sensory cells to effector cells, such as smooth muscle cells, to promote GI motility.^6^

While EECs adjust their function in response to the mechanical cues that surround them, data on basic mechanical properties are lacking entirely. It has become increasingly clear that the mechanical properties of a cell, such as rigidity and viscosity, may offer biophysical markers for determining physiological cell state transitions or pathological cell changes. However, EECs are rare and have been largely inaccessible for *in vitro* studies,^3^ complicating research into the mechanical behavior of these cells. Although few human EEC-immortalized cell lines exist, they differ substantially from their wild-type counterparts.^7^ Adult stem cell (ASC)-derived organoids may provide a solution to this challenge. These organoids represent three-dimensional miniature epithelial structures that can be cultured and expanded outside the body.^8^ These small-scale organs are created from tissue biopsies and consist entirely of primary epithelial cells, leveraging the remarkable expansion capability of adult stem cells. Their unique composition closely mimics the *in vivo* environment, facilitating the study of epithelial physiology and stem cell differentiation dynamics,^3^^,^^9^ as well as human disease.^10^, ^11^, ^12^, ^13^, ^14^, ^15^, ^16^, ^17^, ^18^, ^19^, ^20^ Being derived from human sources, these organoids accurately capture the specific characteristics of human tissues. This collection of features grants them significant power and utility as a valuable research tool.^21^

Here, we report data on the first single-cell mechanical characterization of organoid-derived human ECCs. We used state-of-the-art single-cell optical tweezers to measure the stiffness profiles of EECs. The Clevers group previously generated a high-resolution mRNA and secretome atlas of organoid-derived human EECs.^3^ Herein, TDO2 expression was found in duodenal EECs from the proximal intestine. TDO2 can metabolize tryptophan through the kynurenine pathway and is one of the primary regulators of the availability of this amino acid. Tryptophan is the precursor of serotonin, and TDO2 knockout mice experience increased serotonin levels,^22^ suggesting that TDO2 could locally regulate serotonin production in the gut. We have recently described how cellular metabolism and mechanics are tightly intertwined.^23^ As a proof-of-principle, we, therefore, opted to probe alterations in EEC stiffness profiles following TDO2 inhibition. The presented data on human EECs showcase the new technical possibilities for decoding gut cell mechanics, further contributing to our understanding of how the mechanical properties of cells impact their functioning.

## 2 Materials and methods

### 2.1 Culture and processing of human intestinal organoids

Human small intestinal cells were isolated, processed, and cultured following previously established protocols (Beumer *et al.*, 2018, Sato *et al.*, 2011). In lieu of Wnt-conditioned media, the culture medium was enriched with a Wnt surrogate (0.15 ​nM, U-Protein Express). Organoids were routinely split approximately every 10 days. For this, the organoids were gently separated from the basement membrane extract (BME) using ice-cold AdDMEM/F12 (GIBCO) and mechanically fragmented into smaller pieces using a Pasteur pipette. These fragments were then reseeded in fresh BME.

**Fig. 1:**
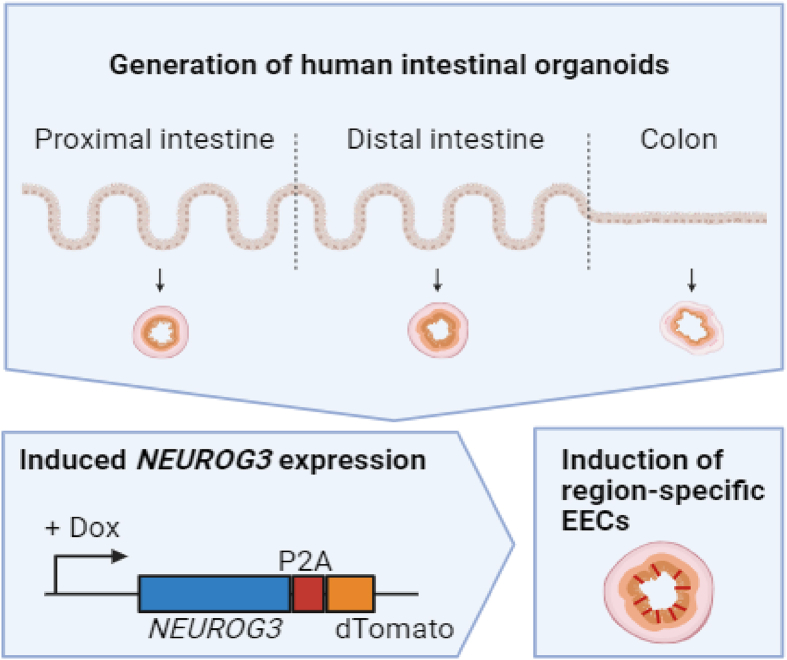
Schematic representation of the generation of region-specific enteroendocrine cells (EECs). Organoids are established from different regions of the intestinal tract of different patients, after which doxycycline (dox)–induced overexpression of neurogenin-3 (NEUROG3) can drive the production of EECs. Created with BioRender.com.

We exploited human organoids harboring an inducible transcription factor, Neurogenin-3 coupled to dTomato, to derive all major EEC lineages.^3^ We next used a previously generated fluorescent reporter for tryptophan hydroxylase 1 (TPH1-mNeongreen), the rate-limiting enzyme in 5-HT synthesis, to isolate a pure population of ECs from these organoids. EECs that were not mNeongreen-positive could be isolated based on dTomato-positivity (Fig. 1). To induce differentiation into enteroendocrine cells (EECs), organoids containing the Neurogenin-3-dTomato inducible system were exposed to 1 ​μg/mL doxycycline (Sigma) in ‘ENR’ medium (Sato *et al.*, 2009) for 48 ​h. Fully mature EECs were achieved after an additional 3-day incubation in ENR without doxycycline. To inhibit tryptophan metabolism, EECs were exposed to 680C91, Tocris, 10 ​μM, for the entirety of EEC differentiation. 680C91 is an inhibitor of tryptophan 2,3-dioxygenase (TDO2), an enzyme that plays a crucial role in tryptophan metabolism by catalyzing the first and rate-limiting step of the kynurenine pathway.

TPH1-reporter organoids have been previously characterized. Organoids were dissociated into single cells by incubating them with TrypLE (TrypLE Express; Life Technologies) for 10 ​min, followed by mechanical disruption through pipetting. Cells were subsequently sorted based on their fluorescence levels using a BD FACS Aria (BD Biosciences), using gating strategies as employed before.

### 2.2 Optical tweezer instrumentation

Our C-trap optical tweezer machine is equipped with an IR laser source with a wavelength of λ ​= ​1064 ​nm implemented in an inverted microscope. The laser power was precisely set to 500 ​mW on each laser beam trapping a 4 ​μm bare silica bead for all measurements. The piezo tracking module of the BlueLake software, commercially available through the manufacturing company (Lumicks, Amsterdam-Zuid, the Netherlands), was used to track the beads at high frequency.

### 2.3 Optical tweezer trap calibration

In order to accurately measure the exerted force, the trap was calibrated before attaching the small bead to the cell. To do so, first, the voltages of the quadrant photodiode (QPD) were recorded for 10 ​s at a sampling rate of 78 ​k Hz. The trap stiffness and the voltage-to-position conversion factor were obtained by power spectrum analysis of the recorded time series. All of these recordings and analyses were automatically processed using the Force Calibration module of the BlueLake software of the C-Trap. Image processing was recorded by a camera and piezo tracking was used for particle positioning at low and high frequencies. It is worth mentioning that the size of the trapped bead was chosen so that the cell was not directly exposed to the laser beam, even in the most displaced position.

### 2.4 Optical tweezers experimental setup and force spectroscopy measurements

**Fig. 2:**
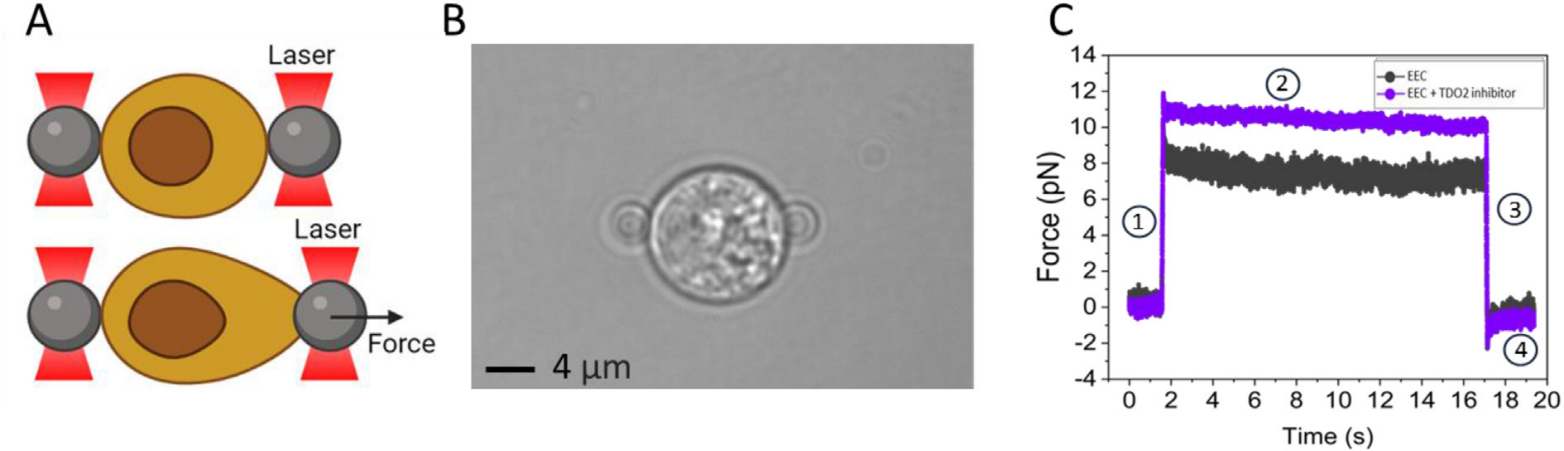
Probing the mechanical properties of EECs using optical tweezers. (A) Schematic representation of the experimental setup. (B) Microscopic image of an EEC trapped between two 4 ​μm beads. (C) Typical trace of force measured as a function of time during a manipulation cycle (10 ​μm/s). The cycle includes four steps: Step 1 (stretch): the EEC is stretched with a pre-known strain rate by moving laser one laser trap with a pre-known velocity, for 500 ​nm. Step 2 (relaxation): the EEC relaxes the exerted tension and reaches mechanical equilibrium for 15 ​s. Step 3: The moved laser trap is returned to its initial position by 500 ​nm at the same velocity used in the first step. Step 4: The sample chamber is kept stationary for over 15 ​s to let the EEC relax, again.

In our optical tweezer experimental setup, EECs isolated from healthy human intestinal organoids were sandwiched between two bare silica beads (≈4 ​μm, silica beads, Bangs-lab). Both beads were first optically trapped after which they were carefully attached on opposite sides of the EEC. To probe the mechanical properties of the EECs, we performed multiple stressing–destressing cycles, in which the cells were pulled and relaxed at a constant strain rate at different values of velocity (1, 5, 10, and 20 ​μm/s). The optical tweezer experimental setup is shown in Fig. 2A. EECs are sandwiched between two optically trapped silica beads and subsequently stretched (Fig. 2B). A typical trace of the force measured as a function of time during a single manipulation cycle (10 ​μm/s) is depicted in Fig. 2C. The manipulation cycle includes the following steps: Step 1 (stretch): the EEC is stretched with a pre-known strain rate by moving laser one laser trap with a pre-known velocity, for 500 ​nm. Step 2 (relaxation): the EEC relaxes the exerted tension and reaches mechanical equilibrium for 15 ​s. Step 3: The moved laser trap is returned to its initial position by 500 ​nm at the same velocity used in the first step. Step 4: The sample chamber is kept stationary for over 15 ​s to let the EEC relax, again.

### 2.5 Quantification and statistical analysis

Only cells showing a distinguished hysteresis loop were included in the analysis. Possible reasons for not obtaining the characteristic loops are the improper attachment of the beads to the cells, cells that are in poor condition (i.e., entered apoptotic phase), and off-centered beads during stretching. All these conditions lead to disrupted hysteresis loops, i.e. loops deviating from a linear regime, making it impossible to determine the slope from the linear ascending part of the stress–strain curve. The normality of the data sets was tested with a Shapiro–Wilk normality test. A student's t-test was used for all data sets to determine significant differences between controls and EECs exposed to TDO2. Data are taken as significant if the p-value is lower than 0.05. For data processing and visualization, Python (version 2.7.15), R (version 3.6.0), and Origin (version 2022) were utilized.

## 3 Results

**Fig. 3:**
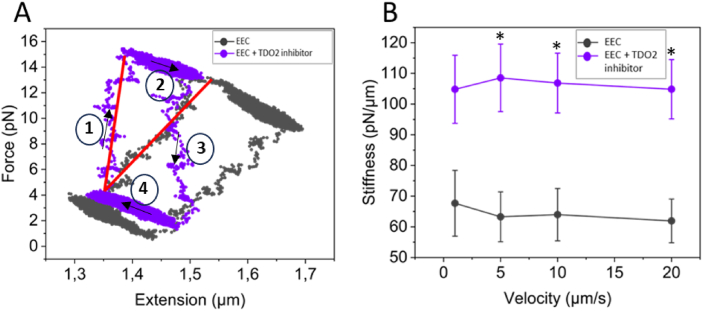
Inhibition of tryptophan metabolism stiffens EECs. (A) Typical hysteresis loops of EECs in the stress–strain curve in response to a force–stressing–destressing cycle (10 ​μm/s) under normal conditions and upon TDO2 inhibition. (B) TDO2 inhibition (n ​= ​11 cells) induces significant stiffening (p ​< ​0.05) of EECs at nearly all stretching velocities, compared to the control (n ​= ​10 cells). Arrows indicate stretch–relax cycles. Data are represented as mean ​± ​SEM. ∗(p ​< ​0.05).

In response to a force–stressing–destressing cycle (10 ​μm/s), EECs show typical hysteresis loops in the stress–strain curve, as shown in Fig. 3A. From the slope of the linear ascending part of the stress–strain curve, the stiffness of the EECs at each stretching velocity can be determined. Average stiffness values for EECs at each stretching velocity are presented in Fig. 3B. It has been increasingly acknowledged that mechanics are intertwined and evolve in parallel with cellular activity. To that end, we have recently described how mechanical cues, sensed through the cytoskeleton or distortion of the cell and organelles, can induce metabolic changes in the cell and how alterations in metabolism, in turn, can feed back to regulate the mechanical properties of cells.^23^ Intriguingly, upon TDO2 inhibition, EECs show significant stiffening (p ​< ​0.05) at nearly all stretching velocities (for 1 ​μm/s p ​= ​0.08), compared to the control.

## 4 Discussion

The coordinated secretion and motility of the GI tract play a pivotal role in ensuring normal digestion. These essential GI functions are finely tuned through orchestrated responses to intraluminal nutrients and mechanical forces. Enterochromaffin cells, the most common type of EECs, are specialized mechanosensors^5^ that in response to mechanical force release serotonin, which stimulates secretory reflexes. While the process of mechanosensation, and the resulting changes in mechanics are essential for EEC functioning, data on basic mechanical properties are lacking. In addition, a growing body of evidence reveals that cellular mechanics are interwoven with other essential cellular processes such as metabolism,^23^ raising the question of how cells might coordinate their responses to biomechanical cues and vice versa. In this study, we addressed these knowledge gaps by probing EEC stiffness using optical tweezers, and its alteration upon exposure to a TDO2 inhibitor, interfering with tryptophan metabolism.

We present the first mechanical characterization of serotonin-producing EECs, exhibiting stiffness values ranging from 60 to 70 ​pN/μm at stretching velocities ranging from 1 to 20 ​μm/s. These stiffness values are a magnitude higher than the stiffness values we previously observed for red blood cells,^24^ and slightly lower than those for primary human monocytes.^25^ In addition, we showed that upon TDO2 inhibition, EECs show significant stiffening. TDO2 plays a pivotal role in the catabolic pathway of tryptophan (an essential building block for proteins and precursor for biologically active compounds such as the neurotransmitter serotonin), where it catalyzes the conversion of tryptophan into kynurenine.^26^ This enzymatic process is the initial and rate-limiting step in the kynurenine pathway. Quinolinic acid (QUIN) is an endogenous downstream metabolite of the kynurenine pathway.^26^ TOD2 inhibition therefore leads to a reduction in QUIN production. QUIN has been shown to disrupt cytoskeletal filaments through hyperphosphorylation.^27^ Lower levels of QUIN might therefore explain the stiffening observed upon treatment with 680C91. However, TDO2 has also been shown to promote cancer progression by mediating tryptophan deprivation and the production of metabolites along the kynurenine pathway. The depletion of tryptophan has been shown to facilitate tumor immune escape by inhibiting the function of T cells.^28^ Furthermore, the metabolites produced in this pathway can provide a source of energy for cancer cells, aiding their growth. Indeed, TDO2 has been shown to promote proliferation, migration, and invasion of various cancer cell types,^29^, ^30^, ^31^, ^32^, ^33^ and especially serotonin-producing neuroendocrine tumors express TDO.^34^ TDO2 inhibition and the associated cell stiffening might therefore offer a potential avenue for reducing gut cancer cell invasiveness and preventing metastasis.

## Ethical approval

This study does not contain any studies with human or animal subjects performed by any of the authors.

## Declaration of competing interest

The authors declare no competing interests related to this work.
